# Stem Cells-Loaded 3D-Printed Scaffolds for the Reconstruction of Alveolar Cleft

**DOI:** 10.3389/fbioe.2022.939199

**Published:** 2022-06-14

**Authors:** Dongyuan Luo, Boying Chen, Yu Chen

**Affiliations:** Guangzhou Women and Children’s Medical Center, Guangzhou, China

**Keywords:** alveolar cleft, β-tricalcium phosphate, methacrylated gelatin, 3D printing, bone marrow mesenchymal stem cells

## Abstract

The advances in the field of tissue engineering and regenerative medicine have opened new vistas for the repair of alveolar clefts. However, the currently available biomaterials used for the repair of alveolar clefts have poor mechanical properties and biocompatibility, which hinders the treatment outcomes. Here, we aimed to develop 3D printed biomimetic scaffolds that fuses β-tricalcium phosphate (β-TCP) and bone marrow mesenchymal stem cells (BMSCs) for improving the repair of alveolar clefts. The methacrylate gelatin (GelMA) was mixed with β-TCP for the preparation of GelMA/β-TCP hybrid scaffolds via 3D printing platform and chemically cross-linking with UV light. The physicochemical properties of the hydrogel scaffolds were characterized. Moreover, the survival state, proliferation ability, morphological characteristics, and osteogenic induction of BMSCs were examined. The prepared hybrid scaffolds showed good biocompatibility and mechanical properties. BMSCs attached well to the scaffolds and proliferated, survived, differentiated, and stimulated osteogenesis for the reconstruction of alveolar clefts. We expect that use of the prepared hybrid hydrogel scaffold can improve the outcomes of alveolar cleft repair in clinic and expand the application of hybrid hydrogel in tissue engineering repair.

## 1 Introduction

Alveolar cleft, which results from a fusion disorder between the embryonic globular process and the maxillary process, is the most difficult challenge in the sequence therapy of cleft lip and palate. Approximately 75% of all patients with cleft lip and palate have concomitant alveolar cleft (Cho-Lee et al., 2013). Effective repair of alveolar cleft can improve the treatment outcomes in patients with cleft lip and palate. The alveolar cleft can be reconstructed either with autologous cancellous tissue or with tissue-engineered scaffolds. The latter approach is associated with relatively higher safety and comfort, by precluding the need for secondary injury to the body. Tissue engineering scaffolds can provide space for cell growth, differentiation, adhesion, and migration in the fissure area. In addition, the ideal scaffolds should exhibit good biocompatibility and degradability, which are beneficial for the growth of osteoblasts and blood vessels. For the reconstruction of alveolar cleft, the scaffold should be good at bone conduction and conducive to certain bone induction. Currently, several materials are used for bone repair, such as metal, hydroxyapatite, and carbon nanotubes. Among these, the hydroxyapatite and other synthetic bio-ceramic tissue-engineered bone scaffolds have been widely used for repair of bone defects (LeGeros, 2002; Klijn et al., 2012). Application of these materials effectively shortens the operation time and precludes the need for cutting the autologous bone, thus reducing the pain and complications. However, the non-deformability and non-biodegradability of these synthetic tissue-engineered bone scaffolds hinder the tooth eruption and orthodontic treatment (Feinberg et al., 1989; Chin et al., 2005). Especially during the bone healing period, the scaffolds tend to shift when exposed to stress (Letic-Gavrilovic et al., 2003). Therefore, they are not a suitable base for the movement of teeth and reconstruction of dental arch. Further research is required to develop biological absorbable scaffolds for bone reconstruction of alveolar cleft.

Biological 3D printing technology has been widely used in tissue repair engineering due to its high controllable accuracy and simple operation. In the field of tissue engineering, biological 3D printing technology can accurately control the morphology of scaffold material and the distribution of cells inside scaffolds. Moreover, the technology can enable construction of artificial tissues, which are highly similar to the human tissue structure. These structures show distinct advantage over previous scaffolds. 3D printing technology ensures that the printed scaffolds have the optimal anatomical configuration for *in-situ* implantation. Therefore, biological 3D printing technology has been applied in personalized medical models, permanent human implants, bionic scaffolds, drug testing models, controllable and sustained-release drugs, and other aspects, promoting the continuous development of biomedicine. Besides the bioactive tissue scaffolds, functional cells are required for alveolar cleft repair, so as to accelerate the reconstruction. Stem cells have been used in the treatment of a variety of diseases. Several reports have described the application of stem cells in alveolar cleft. Therefore, combining three-dimensional scaffolds with suitable stem cells is expected to have a good repair effect (Bajestan et al., 2017). Stem cells derived from a wide range of sources, such as bone marrow-derived stem cells (BMSCs) (Sousa et al., 2014), adipose tissue-derived stem cells (Benazzo et al., 2014), embryo-derived stem cells (Liu et al., 2014), and dental tissue-derived stem cells (Hilkens et al., 2014) have been used in the repair of alveolar cleft. Currently, BMSCs are being widely used in bone tissue engineering and bone defect repair due to the ability of proliferation and multidirectional differentiation potential. Thus, they can induce stable bone tissue formation *in vivo*, with no obvious bone absorption phenomenon. Moreover, these can help achieve sustained treatment effect for a long time (Middleton and Tipton, 2000; Ramakrishna et al., 2004; Hibi et al., 2006; Gimbel et al., 2007).

In this study, we developed 3D hydrogel scaffolds composed of GelMA and β-TCP. We determined the mechanical properties of GelMA/β-TCP scaffolds through electronic universal materials testing machine and then observed the microstructure of freeze-dried hydrogel scaffolds by scanning electron microscopy (SEM). The results showed good biocompatibility and biomechanical properties of hydrogel, which can contribute to the adhesion, proliferation, and differentiation of cells *in vivo*. The interaction between BMSCs and GelMA/β-TCP scaffolds was investigated. We found that the BMSCs adhered well to the hydrogel scaffold, and showed normal cell morphology, proliferation, and differentiation. With the rapid advances in 3D printing technology and tissue engineering, development of 3D printing materials loaded with stem cells for alveolar cleft bone grafting is of great clinical significance. Leveraging the two technologies can help address problems such as insufficient control of accuracy of scaffold structure, randomness of stem cell distribution, and weak mechanical strength. Our results have important implications for the application of 3D GelMA/β-TCP hybrid scaffolds in alveolar cleft bone grafting.

## 2 Materials and Methods

### 2.1 Preparation of 3D GelMA/β-TCP Scaffolds

#### 2.1.1 Printing of GelMA Scaffolds

Briefly, 5 g of GelMA was dissolved into 100 ml ultrapure water in a 37°C water bath. The GelMA solution was placed into the 20 ml steel syringe and then transferred to 4 °C for 30 min. The steel nozzle was maintained at 26°C and placed into the syringe for another 30 min. The temperature of printer platform was maintained at 3°C. Hydrogel microfilaments were dispensed to the base plate through the nozzle by applying pneumatic pressure of 360 kPa. The mechanical parameters were set at the crosshead velocity of 360 mm/min using 25 G nozzle. The distance between nozzle and surface of base plate was maintained around 0.2 mm. Subsequently, the scaffolds were solidified by illumination with the light source of 3W at 405 nm for 50 s. The GelMA scaffolds with dimensions of 10 × 10 × 2 mm^3^ were fabricated.

#### 2.1.2 Printing of GelMA/β-TCP Scaffolds

β-TCP was mixed into the 5% GelMA (w/v) solution to achieve final concentration of 1% β-TCP (w/v) at 37 °C. The mixture of GelMA/β-TCP solution was placed into a 20 ml steel syringe and then transferred to 4°C for 30 min. The steel nozzle was maintained at 25°C and placed into the syringe for another 30 min. Meanwhile, the temperature of printer platform was adjusted to 3°C. Hydrogel microfilaments were dispensed to the base plate through the nozzle by applying pneumatic pressure of 360 kPa. The mechanical parameters were set at the crosshead velocity of 360 mm/min using 25 G nozzle. The distance between nozzle and surface of base plate was maintained around 0.2 mm. Subsequently, the scaffolds were solidified by illumination with the light source of 3W at 405 nm for 50 s. The GelMA/β-TCP scaffolds with dimensions of 10 × 10 × 2 mm^3^ were fabricated.

### 2.2 Characterization of GelMA/β-TCP Scaffold

Cylindrical hydrogel of 8.8 mm diameter and 5.5 mm height were placed between the parallel plates in the electronic universal materials testing machine for the detection of Young’s modulus. The strain rate was controlled for 15% per minute, while the cylindrical hydrogel deformation varied from 0% to 75%. The changes in the stress and strain monitored by the machine were recorded. The slope of the strain segment in the first 10% of the stress-strain curve was the hydrogel compression modulus.

### 2.3 Morphological Analysis of GelMA/β-TCP Scaffold

The morphology of the fabricated GelMA/β-TCP scaffolds was examined by SEM. Before the detection, the hydrogels were flash-frozen in the refrigerator at -80°C. Then, the samples were cut into 2 × 2 mm^2^ with the scalpel. Subsequently, the small samples were transferred to be lyophilized. Finally, the treated hydrogels were fixed on the special stubs for examination by SEM.

### 2.4 Effect of GelMA/β-TCP on Proliferation of BMSCs

For evaluation of BMSCs proliferation, 0.5 ml of 5% GelMA, 5% GelMA +1%β-TCP, and 5% GelMA +5% β-TCP were added to the 12-well plate and solidified by illumination with the light source of 3W at 405 nm for 50 s as described above. The *in-situ* hydrogels were soaked in 75% alcohol for 2 h and then supplemented with DMEM/F12 complete medium overnight. BMSCs were inoculated in each well at the density of 1×10^5^ cells/mL. There were four parallel groups in the experiment. The cell proliferation was assessed with the addition of 50 μL CCK8 reagent at days 1, 4, 7, and 10 for 3 h. Subsequently, the cells were detected by microplate reader (absorbance: 450 nm). The proliferation of BMSCs was assessed by the OD450 values.

### 2.5 Effect of GelMA/β-TCP on the Viability of BMSCs

For evaluation of the viability of BMSCs in the GelMA/β-TCP scaffold, the scaffolds were treated with alcohol and DMEM/F12 medium according to the procedure described above. Subsequently, the scaffolds were placed in 12-well plates, and each well was inoculated with 1×10^4^ BMSCs for continuous culture of 10 days. The GelMA/β-TCP scaffolds were removed after inoculation of 1 and 10 days for dead/alive staining analysis. 2 µM of calcein AM and 8 µM of propidium iodide (PI) were mixed for preparation of the working solution. The scaffold was washed thrice with 1×PBS. Then the prepared working solution was added and incubated with the cells for 45 min at room temperature. After incubation, the scaffolds were washed again with 1×PBS. Anti-fluorescence quenching agent was added into the samples and the samples were imaged by Olympus FV3000 scanning laser confocal microscope. Green fluorescence indicated the alive cells marked by the calcitanin AM, while red fluorescence indicated the dead cells marked with PI.

### 2.6 Effect of GelMA/β-TCP on the Morphology of BMSCs

For assessing the morphology of BMSCs in the GelMA/β-TCP scaffold, the scaffolds were treated with alcohol and DMEM/F12 medium as described above. Subsequently, the scaffolds were placed in 12-well plates, and each well was inoculated with 1×10^4^ BMSCs for continuous culture of 10 days. The GelMA/β-TCP scaffolds were removed after inoculation for 10 days for cytoskeleton staining analysis. The scaffold was washed thrice with 1×PBS and immersed in 4% paraformaldehyde for 0.5 h and then washed 3 times. Then, 0.5% TritonX-100 was added into the scaffolds for 10 min. The washing operations were repeated as above. Subsequently, the cells loaded hydrogels were immersed into the 1 mg/ml phalloidine for 0.5 h and then transferred to the 1 μg/ml DAPI for 10 min. After staining of antibody, anti-fluorescence quenching agent was added to the scaffolds and stored at 4°C. For analysis of immunofluorescence staining results, images were collected by Olympus FV3000 scanning laser confocal microscope and analyzed for growth of BMSCs.

### 2.7 Effect of GelMA/β-TCP on Osteogenic Differentiation of BMSCs

For evaluation of osteogenic differentiation, the BMSCs loaded scaffolds were fixed with 4% paraformaldehyde for 15 min at room temperature. Subsequently, the scaffolds were washed thrice with 1×PBS. Subsequently, the scaffolds were stained with ALP dyes. BCIP/NBT is the common substrate for alkaline phosphatase. BCIP can be hydrolyzed to a highly reactive product that reacts with NBT to form an insoluble NBT-Formazan of dark blue or blue-purple color. The scaffolds were incubated with the dye at room temperature for 2 h. Then, the dyeing solution was removed and washed twice with distilled water to stop the color reaction. The cells were imaged by light microscopy.

## 3 Results

### 3.1 Characterization of Hydrogel Scaffolds

**FIGURE 1:**
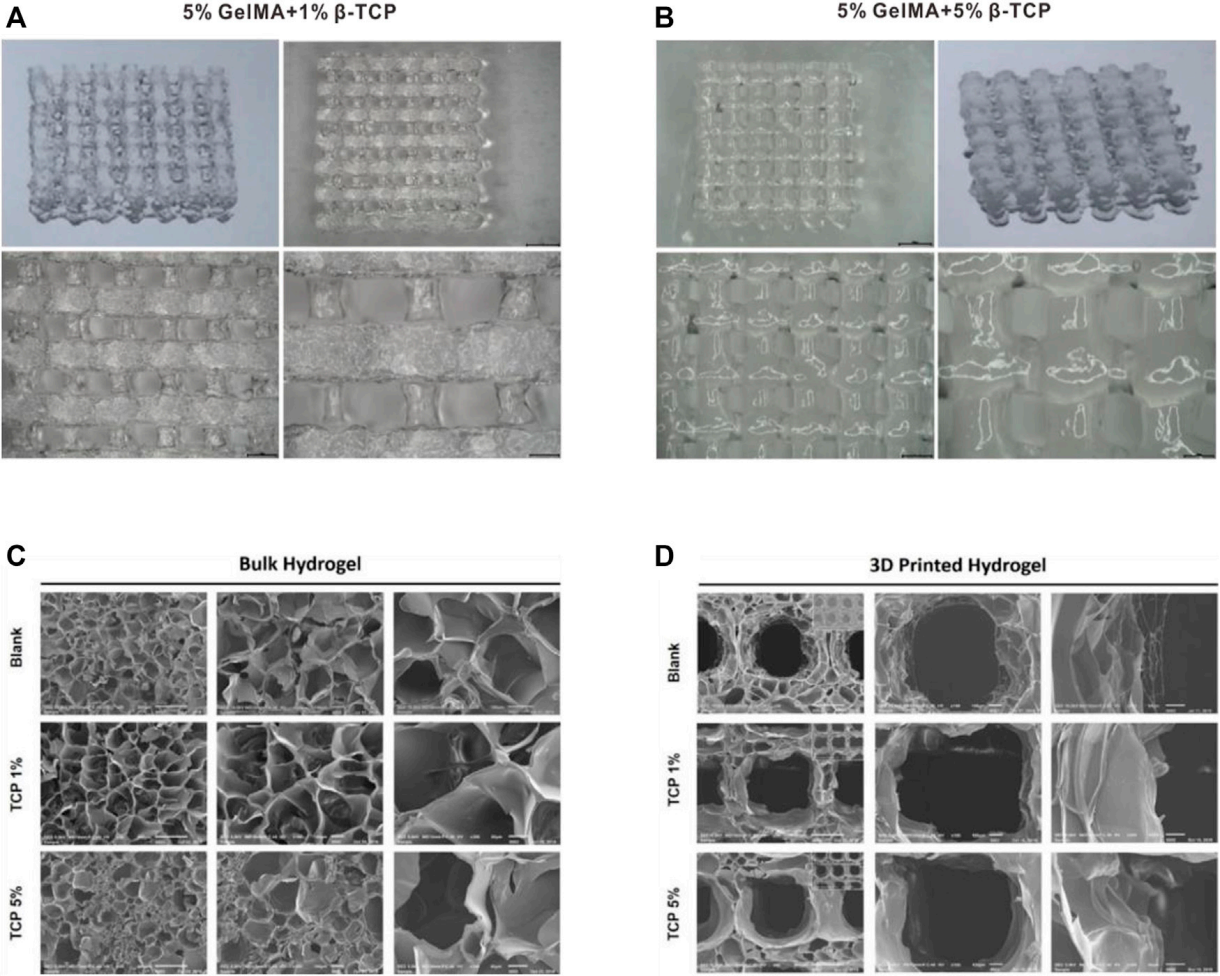
Morphological characteristics of gel scaffolds. Morphology of hydrogels composed of 5% GelMA (w/v) with 1% β-TCP (w/v) **(A)** and 5% GelMA (w/v) with 5% β-TCP (w/v) **(B)**. Lyophilized hydrogels observed by scanning electron microscopy **(C)**. 3D printed hydrogel scaffolds observed by scanning electron microscopy **(D)**.

We successfully prepared GelMA scaffolds via the 3D printing platform according to the methods described above. For improving the bio-absorbability and biomechanical properties, β-TCP was added into the reaction system to fabricate the hybrid GelMA/β-TCP hydrogel scaffolds. The microstructure of 5% GelMA (w/v) with 1% β-TCP (w/v) and 5% GelMA (w/v with 5% β-TCP (w/v) scaffolds was examined and the difference of stiffness and mesh pore size was investigated. The microstructure of 5% GelMA (w/v) with 1% β-TCP (w/v) scaffolds exhibited clear meshes, uniform pores, and uniform wire diameters (Figure 1A). The width of mesh pore ranged from 474 to 929 μm. The microstructure of 5% GelMA (w/v) with 5% β-TCP (w/v) scaffolds was similar with mesh pore widths ranging from 437 to 673 μm (Figure 1B). To further confirm the inner structure of hybrid scaffolds, we examined the microstructure of different hydrogels using SEM (Figure 1C). The pore wall of complete GelMA hydrogel was found to be smooth, and the pore wall roughness was increased with the addition of β-TCP from 1% to 5%. Some granular bulges on the smooth pore wall of the GelMA/β-TCP hydrogel were observable. The printed 3D hydrogel scaffolds freeze-dried were observed by scanning electron microscopy (Figure 1D). As shown in the results, the mesh aperture of Blank group was approximately 500–600 μm. By contrast, the mesh aperture of hybrid GelMA/β-TCP was approximately 600–800 μm. These results suggested that 3D hydrogel scaffolds fused with β-TCP are more conducive to the attachment and growth of stem cells.

### 3.2 Mechanical Properties of GelMA/β-TCP Hydrogels

**FIGURE 2:**
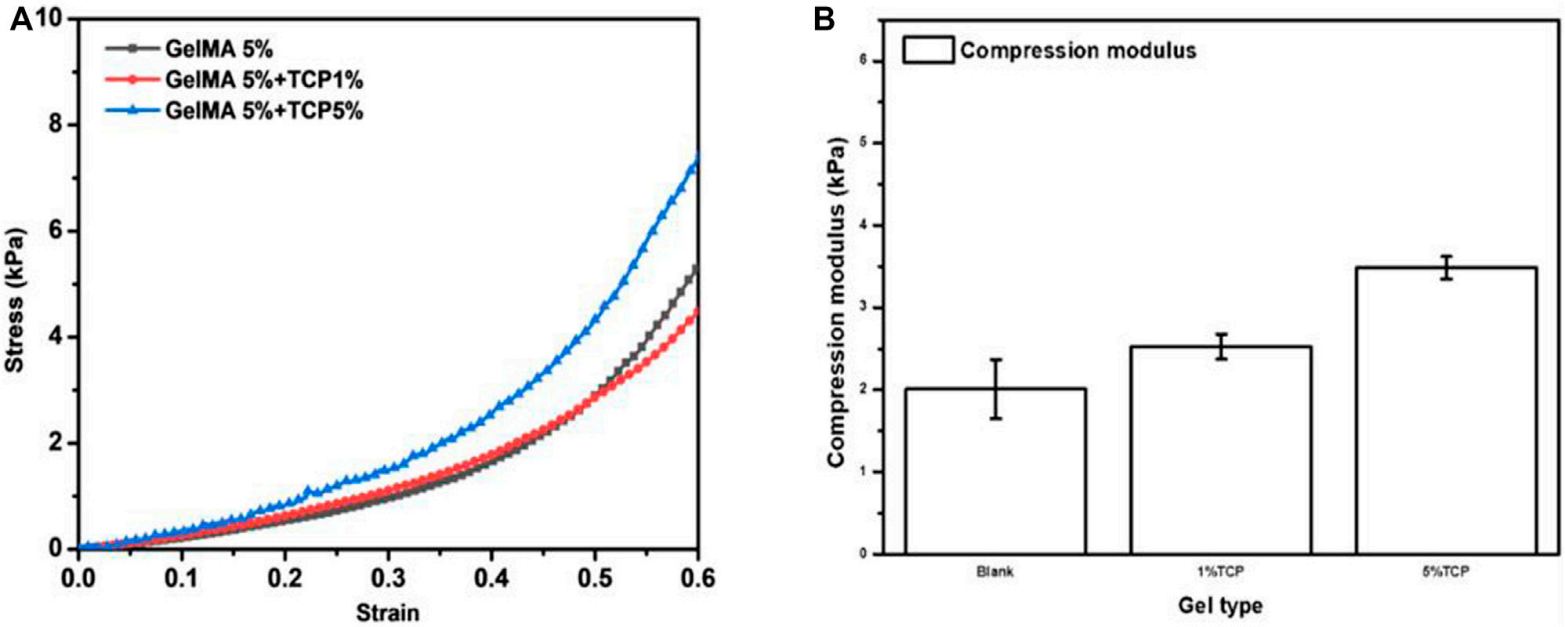
Mechanical properties of GelMA/β-TCP hydrogels **(A)** The stress-strain curves of different hydrogels. **(B)** Anti-stress modulus of different hydrogel calculated by the changes in stress to strain (n = 4).

The above results showed that we were able to print a relatively ideal structural morphology; however, this was far from enough for the use of biological scaffolds *in vivo*. Biological scaffolds must also have excellent biomechanical properties. Therefore, the mechanical properties of GelMA/β-TCP hydrogel were investigated. The anti-stress properties of GelMA, 5% GelMA (w/v) with incorporation of 1% β-TCP (w/v) and 5% GelMA (w/v) with incorporation of 5% β-TCP (w/v) hydrogels were tested. As shown in Figure 2A, the anti-stress ability of 5% GelMA (w/v) with the addition of 5% β-TCP (w/v) hybrid hydrogels was significantly greater than that of GelMA and 5% GelMA (w/v) with the addition of 1% β-TCP (w/v) hydrogel. When the tension was less than 0.5, there was no difference in the anti-stress strength of GelMA and 5% GelMA (w/v) with the addition of 1% β-TCP (w/v) hybrid hydrogels; however, when the tension exceeded 0.5, the anti-stress strength of GelMA was stronger than that of 5% GelMA (w/v) with the addition of 1% β-TCP (w/v). Moreover, we assessed the mechanical properties of the three different hydrogels. As shown in Figure 2B, the addition of β- TCP increased the anti-stress properties of hybrid hydrogels. These results suggested that the addition of β-TCP can improve the biomechanical properties of hydrogel scaffolds and contribute to its application for *in vivo* tissue repair.

### 3.3 Survival of BMSCs on the Surface of GelMA/β-TCP Hydrogels

**FIGURE 3:**
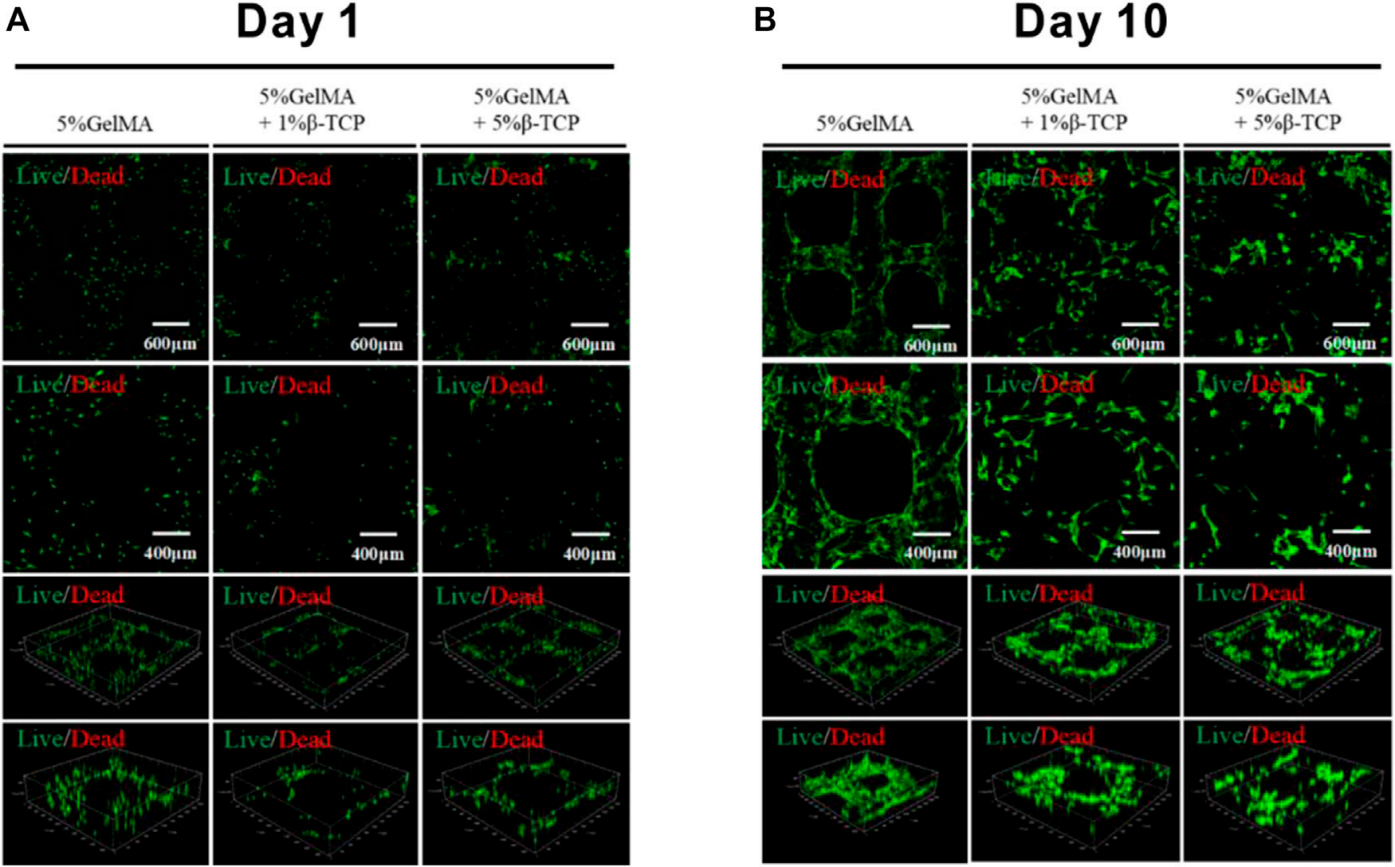
Viability of BMSCs attached to the surface of hydrogel scaffolds. The surviving BMSCs attached to the different scaffolds were identified through immunofluorescence staining after incubation for 1 day **(A)** or 10 days **(B)**.

After successful preparation of the hybrid hydrogel scaffolds with uniform morphology and excellent bio-mechanical performance, we assessed whether BMSCs can survive on the surface of the scaffolds. BMSCs sourced from mice were incubated with the scaffolds. The survival of BMSCs was determined 1 day and 10 days after the inoculation, respectively. The survival rate of BMSCs was close to 100% after 1-day inoculation (Figure 3A). Further, the cells were evenly distributed on the surface of the scaffolds. After 10 days, the scaffolds attached with BMSCs were taken out for immunofluorescence staining with the AM/PI kit. As shown in Figure 3B the cell survival rate was >95%. Compared to the GelMA group, the BMSCs in the 5% GelMA with 1% β-TCP group and the 5% GelMA with 5% β-TCP group were more densely distributed. These results confirmed that the prepared scaffolds did not influence the activity of the attached cells.

### 3.4 Proliferation of BMSCs on the Surface of GelMA/β-TCP Hydrogels

**FIGURE 4:**
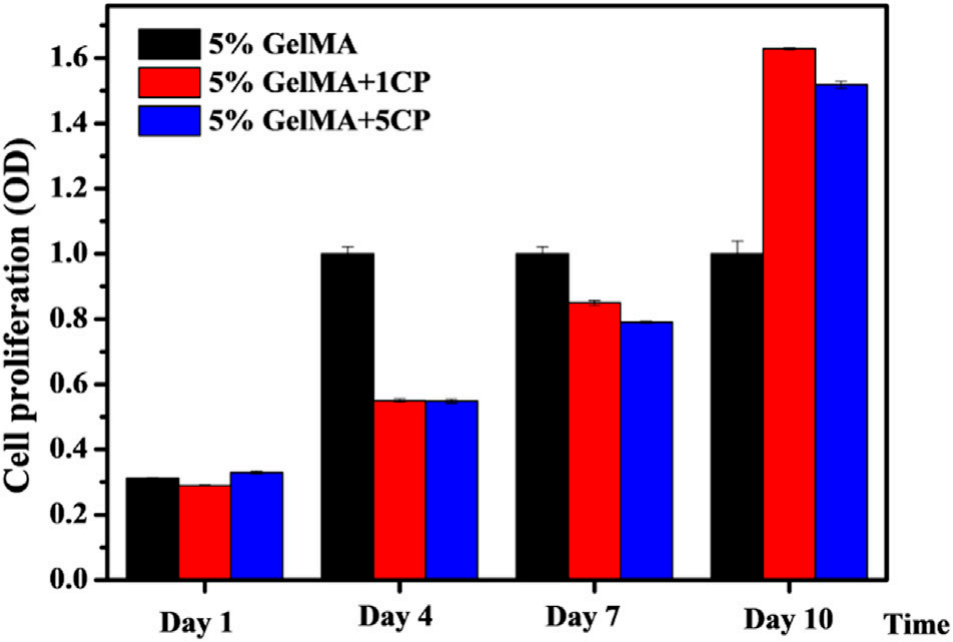
Proliferation of rat BMSCs attached to the surface of GelMA/β-TCP hydrogel scaffolds. BMSCs were incubated into hydrogel scaffolds for culture. Cell proliferation was assessed from day 1 to day 10 using CCK8 assay (n = 4).

In order to reconstruct alveolar cleft, bionic scaffolds need to be able to induce bone, guide bone, and induce osteogenic differentiation of stem cells. Therefore, we explored the proliferation of rat BMSCs induced by the prepared hybrid scaffold. As shown in Figure 4, there was no significant difference in the proliferation of BMSCs on the surface of GelMA scaffolds and GelMA/β-TCP hybrid hydrogel scaffolds after 1 day. We also assessed the proliferation of BMSCs on day 4 and day 7. It was found that the proliferation of rat BMSCs attached on the surface of GelMA scaffolds was better than that of the other GelMA/β-TCP hybrid hydrogel scaffolds. However, after 10 days, the proliferation of rat BMSCs attached on the surface of GelMA/β-TCP hybrid hydrogel scaffolds was much better than that of GelMA scaffolds. These results suggested that with the extension of culture time, the GelMA/β-TCP hybrid hydrogel scaffolds were more conducive to the adhesion and proliferation of rat BMSCs. These findings indicate the suitability of hybrid hydrogel scaffolds fabricated by the GelMA/β-TCP for *in vivo* application for alveolar cleft reconstruction.

### 3.5 Morphology of Rat BMSCs Attached to the Surface of GelMA/β-TCP Hydrogel Scaffolds

**FIGURE 5:**
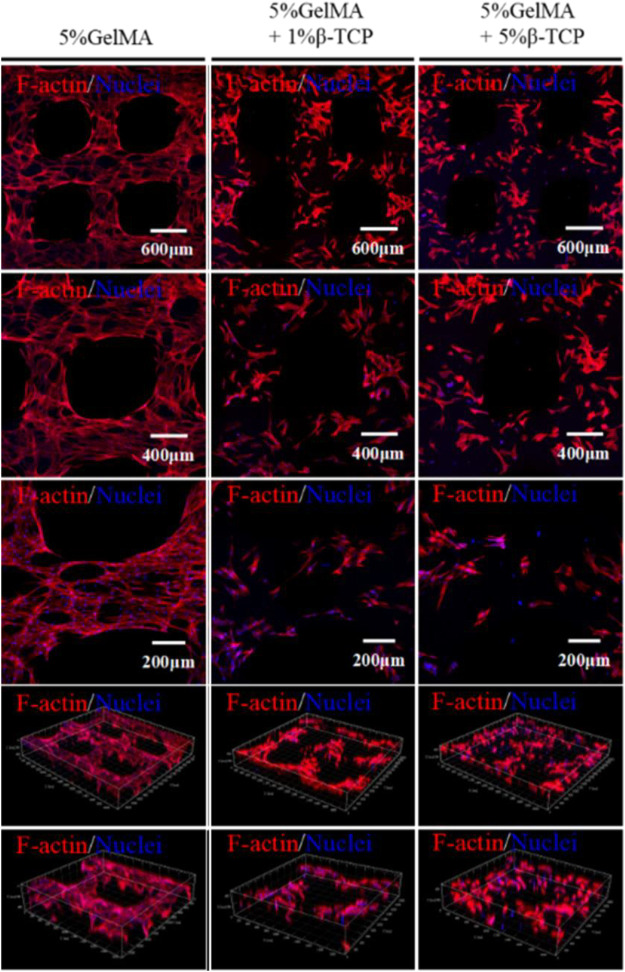
Cytoskeleton structure of rat BMSCs attached to the surface of the GelMA/β-TCP hydrogel scaffolds. After continuous culture of 10 days, the GelMA/β-TCP hydrogel scaffold loaded with BMSCs was taken out for cytoskeleton staining with Rhodamine labeled Phalloidin, and nuclear staining with DAPI. Images were captured by scanning laser confocal microscope.

The above experiments demonstrated that BMSCs could adhere to the hybrid hydrogel scaffolds and proliferate. To further verify that BMSCs were not abnormal proliferators, the morphology of rat BMSCs attached to the surface of GelMA/β-TCP hydrogel scaffolds was observed by fluorescence microscopy. When the GelMA/β-TCP hydrogel scaffolds loaded with BMSCs were cultured continuously for 10 days, the scaffold was removed for immunofluorescence staining of the skeleton. The coverage rate of BMSCs in the 5% GelMA with 1% β-TCP and 5% GelMA with 5% β-TCP groups was found to be lower than that in the 5% GelMA group (Figure 5). In addition, the morphology of the skeletons showed differences between them. We hypothesized that such differences were due to some biological behaviors such as cell differentiation.

### 3.6 Osteogenic Induction of Rat BMSCs on the Surface of GelMA/β-TCP Hydrogel Scaffolds

**FIGURE 6:**
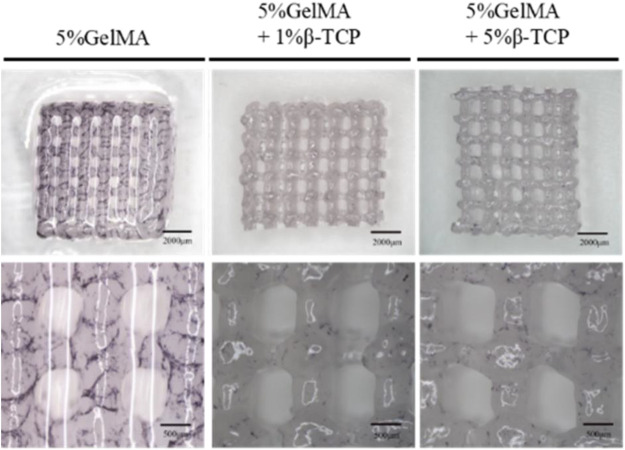
Osteogenic ability of rat BMSCs attached to the surface of the GelMA/β-TCP hydrogel material. The hybrid hydrogel scaffolds loaded with BMSCs were cultured continuously for 21 days. ALP reaction was performed in the scaffolds, and then images were captured by optical microscope.

To further illustrate the influence of hydrogel scaffolds on the osteogenic ability of BMSCs in each group, the fabricated hydrogel scaffolds loaded with BMSCs were continuously induced and cultured for 21 days. Subsequently, the scaffolds were removed and subjected to alkaline phosphatase staining. Alkaline phosphatase expression was obvious in 5% GelMA group after 21 days (Figure 6). Lesser expression was found in 5% GelMA with 1% β-TCP and 5% GelMA with 5% β-TCP groups. These results indicated that the hydrogels composed of GelMA and β-TCP were inferior to GelMA hydrogel in terms of osteogenic induction ability, but the hydrogels fabricated with GelMA and β-TCP still exhibited partial osteogenic induction ability. These results showed that although the prepared GelMA/β-TCP hybrid hydrogel scaffolds showed limited osteogenic induction, it was sufficient for the reconstruction of alveolar cleft.

## 4 Discussion

Reconstruction of alveolar cleft is an important step in the sequential treatment of cleft lip and palate. However, there are still many controversies about the treatment of alveolar cleft. In the 1970s, Boyne and Sands (Boyne and Sands, 1972; Boyne and Sands, 1976) proposed the use of autologous cancellous bone for reconstruction and repair of alveolar cleft. In addition, they also suggested that bone grafting must be performed with autologous iliac cancellous bone before the emergence of cusp teeth in order to achieve good clinical results. Therefore, grafting of iliac cancellous bone mixed with dentition stage to repair alveolar cleft is widely used (Murthy and Lehman, 2005). Tissue regeneration engineering has developed rapidly in recent years. Tissue engineering scaffolds have been widely used as candidate graft materials for alveolar fissure reconstruction. This help reduce the occurrence of pain and complications by precluding the need for cutting the body bone. However, most tissue-engineered scaffolds have exhibited non-absorbability and non-deformability, which hinder tooth eruption and bone movement required for orthodontic treatment (Feinberg et al., 1989; Chin et al., 2005). Moreover, they are prone to shift when stressed during healing (Letic-Gavrilovic et al., 2003), failing to provide appropriate matrix for tooth movement and reconstruction of the dental arch. Therefore, development of bio-absorbable scaffold material with bone inducible activity is a key imperative for repair of alveolar cleft.

Based on the disadvantages of the bio-materials mentioned above, we sought to identify new materials that can be induced for bone induction. The GelMA hydrogel was first reported by Van den Bulcke in 2000 (Van Den Bulcke et al., 2000). GelMA hydrogel is mainly made up of methylacrylamide with Irgacure 2959 as light initiator, and the GelMA hydrogel is prepared by the illumination of UV light. The GelMA hydrogel has been successfully applied for the development of 3D vascular network. For example, Chen (Chen et al., 2012) and Lin (Lin et al., 2013) showed that endothelial cluster cells (ECFC) and mesenchymal stem cells (MSCs) loaded GelMA hydrogel can be used as attached stent for the study of vascular formation *in vivo* and *in vitro*. Moreover, the application of 3D printing technology allows fabrication of GelMA hydrogel with unique morphology, patterns, and 3D structure, which facilitates the provision of nutrients and oxygen to support the surrounding tissue cells and the removal of metabolic products. However, the mechanical performance of GelMA hydrogel is very poor, which is another key point in the design of scaffolds materials. The β-calcium phosphate (β-TCP) is a bio-ceramic that has the potential to improve biomechanical properties and can be used for the preparation of engineered bones via 3D printing techniques (Ma et al., 2019; Beheshtizadeh et al., 2021; Kaimonov et al., 2021). Here, we sought to combine the best of GelMA hydrogel and β-TCP and adjust the ratio of the two to achieve optimal performance of osteogenic scaffolds. We identified and prepared three different scaffolds of 5% GelMA without β-TCP, 5% GelMA with 1% β-TCP, and 5% GelMA with 5% β-TCP. Their microstructures were examined by SEM. We found that the printed structure of different scaffolds was clear with uniform pore size. Further the width of the grids was moderate, which is beneficial for the attachment of stem cells. In addition, we also explored the mechanical load performance of different hydrogels, and found that the anti-pressure performance of hydrogels improved with increase in the β-TCP ratio.

The affinity of BMSCs and hybrid hydrogels determines the biological activity of scaffolds for the reconstruction of alveolar cleft. We inoculated BMSCs into the hydrogel scaffolds for culture. After 10 days, the BMSCs were found to have attached on the hydrogel scaffolds. The cell survival rate was approximately 95%. Moreover, with the extension of incubation time, the hybrid gels were more conducive to the adhesion and proliferation of BMSCs. In addition, osteogenic ability is an important functional indicator of gel scaffolds. After 21 days of culture, the cells were analyzed by alkaline phosphatase staining. The results demonstrated the osteogenic ability of GelMA/β-TCP hybrid gels, which is important for the reconstruction of alveolar cleft.

In conclusion, we successfully developed novel graft materials of GelMA/β-TCP for alveolar cleft reconstruction. In our preliminary experiment, the 3D hybrid hydrogel materials of GelMA/β-TCP exhibited the basic properties required for the reconstruction of alveolar cleft. It was found to induce osteogenic differentiation of BMSCs. We believe that the technique can help improve the outcomes of clinical alveolar cleft reconstruction and expand the application of hydrogel scaffolds in the field of tissue engineering repair.

## Data Availability

The raw data supporting the conclusion of this article will be made available by the authors, without undue reservation.
